# Thoracic Endometriosis: Still a Diagnostic Dilemma

**DOI:** 10.7759/cureus.15610

**Published:** 2021-06-12

**Authors:** Madhu Singh, Rahul B Singh, Abhishek B Singh, Aziel L Carballo, Ayushi Jain

**Affiliations:** 1 Obstetrics and Gynecology, Dr. Balwant Singh's Hospital Inc, Georgetown, GUY; 2 Accident and Emergency, Dr. Balwant Singh's Hospital Inc, Georgetown, GUY; 3 Internal Medicine, Dr. Balwant Singh's Hospital Inc, Georgetown, GUY; 4 Radiology, Mayo Clinic, Jacksonville, USA

**Keywords:** thoracic endometriosis, right sided pleural effusion, haemorrhagic pleural effusion, catamenial hemothorax, video-assisted-thoracoscopy

## Abstract

We report a case of thoracic endometriosis syndrome (TES) presenting with a five-week history of progressive shortness of breath, cough, and wheezing. Investigations revealed a large, right-sided pleural effusion that was bloody on aspiration. A diagnosis of TES was one of the diagnoses entertained and eventually confirmed on finding evidence of pelvic endometriosis on laparotomy. The management of TES should include hormonal therapy, surgical management, or a combination of both.

## Introduction

Thoracic endometriosis syndrome (TES) represents a rare, underdiagnosed clinical entity caused by the presence of endometriotic tissue in the lungs or pleural space. TES is a difficult entity to diagnose due to its non-specific presenting features, such as pneumothorax [1], and the difficulty in establishing the temporal relationship with menses [2]. The difficulty in identifying TES can lead to significant delays before patients are treated appropriately [3]. Following diagnosis and the initiation of therapy, most patients with TES will still experience a recurrence of symptoms within one year [1]. Thus, early diagnosis and treatment of TES can be crucial to reducing morbidity. This case report highlights the dilemmas one may encounter when managing a patient with TES.

## Case presentation

A 38-year-old woman presented to the emergency department with progressive shortness of breath, cough, and wheezing for the last five weeks. One week prior, she presented to another hospital with dyspnea on mild exertion and was advised to return for a chest X-ray if her shortness of breath did not improve. The patient had a past history of dysmenorrhea for several years, along with a history of abdominal pain and fibroids. Her last menstrual period was one week prior to presenting to us.

On examination, decreased breath sounds were noted over the base of her right lung, and harsh breath sounds were noted bilaterally. After evaluating the patient in the emergency department, the patient was advised admission. However, the patient declined to be admitted and preferred to be managed as an outpatient the following day. When the patient returned the next day, she was diagnosed with a right-sided pleural effusion and ascites. Consequent to the detection of the pleural effusion, the patient was again advised admission, to which she agreed.

Investigations

Prior to admission, an abdominal ultrasound showed an enlarged uterus, with fibroids and a moderate amount of free fluid in the abdomen and pelvis. A gross amount of fluid with diffuse internal echoes in the right pleural cavity was seen as well.

**Figure 1:**
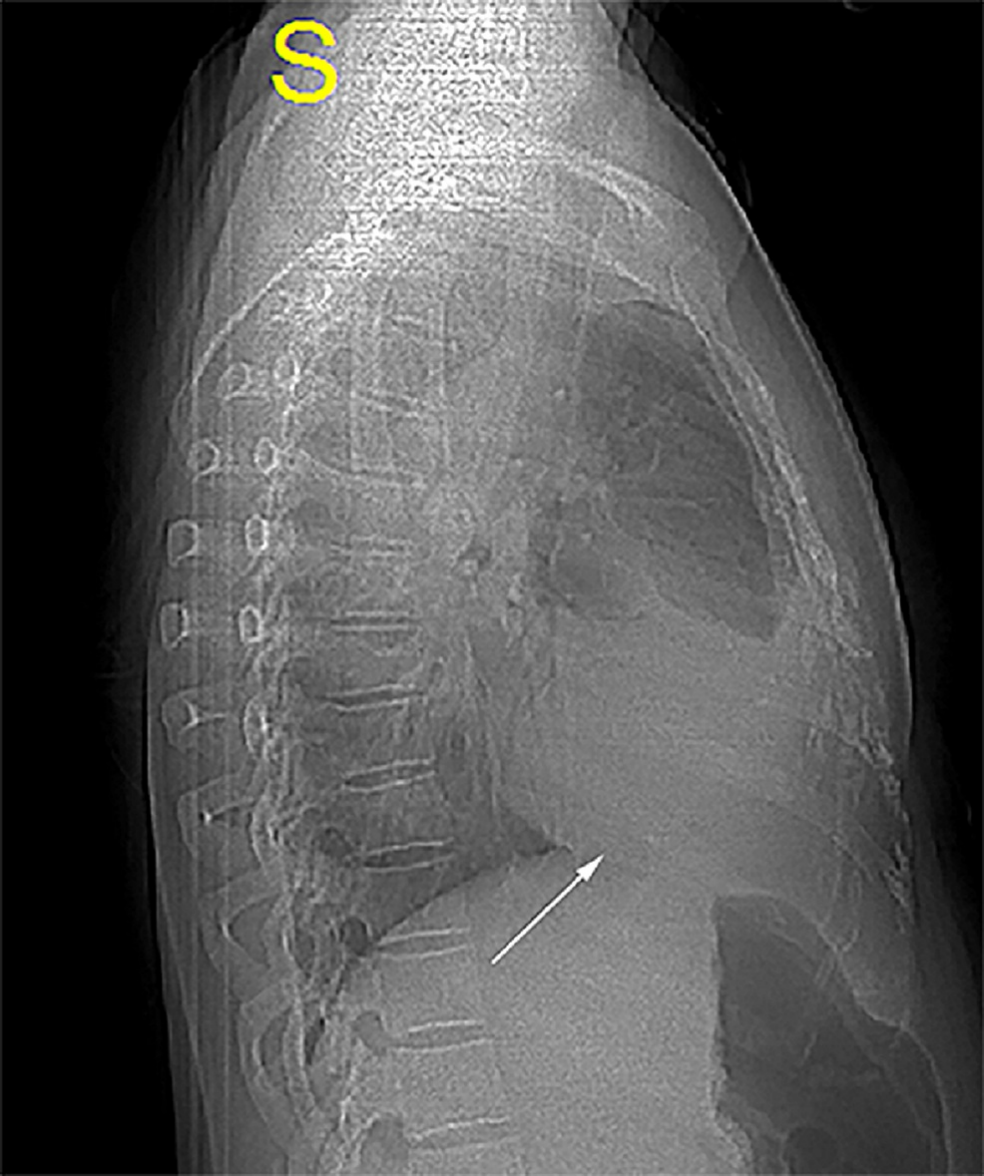
Chest X-ray (right lateral view) showing the extent of the right pleural effusion

**Figure 2:**
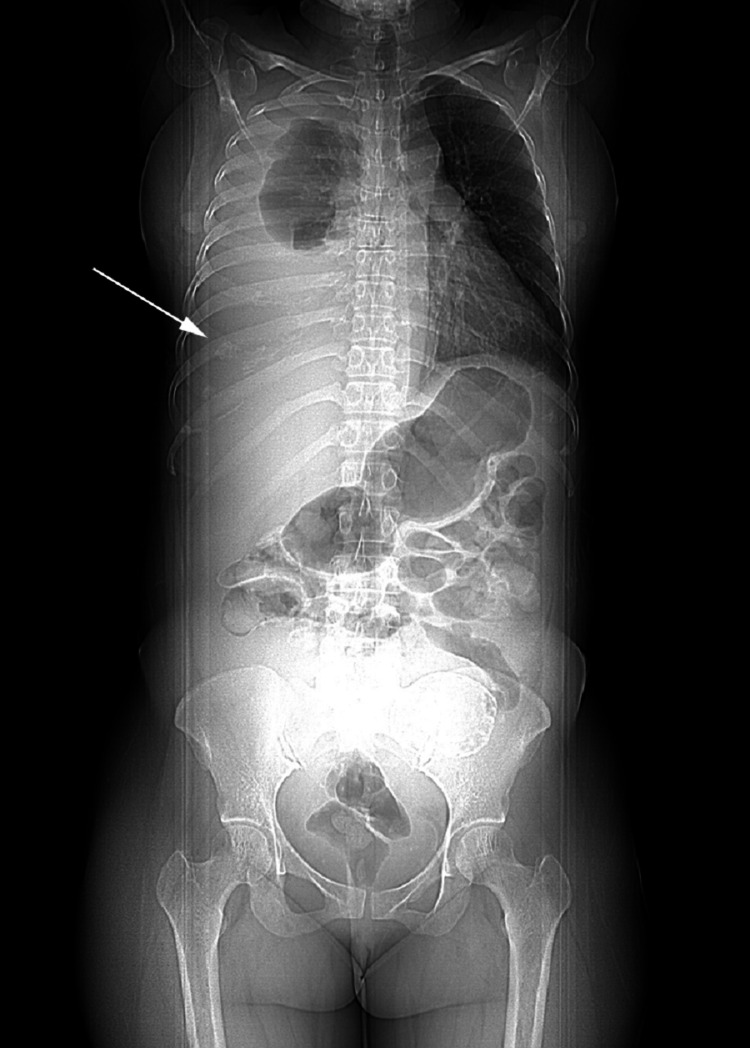
Chest X-ray taken in the supine position showing the effusion

**Figure 3:**
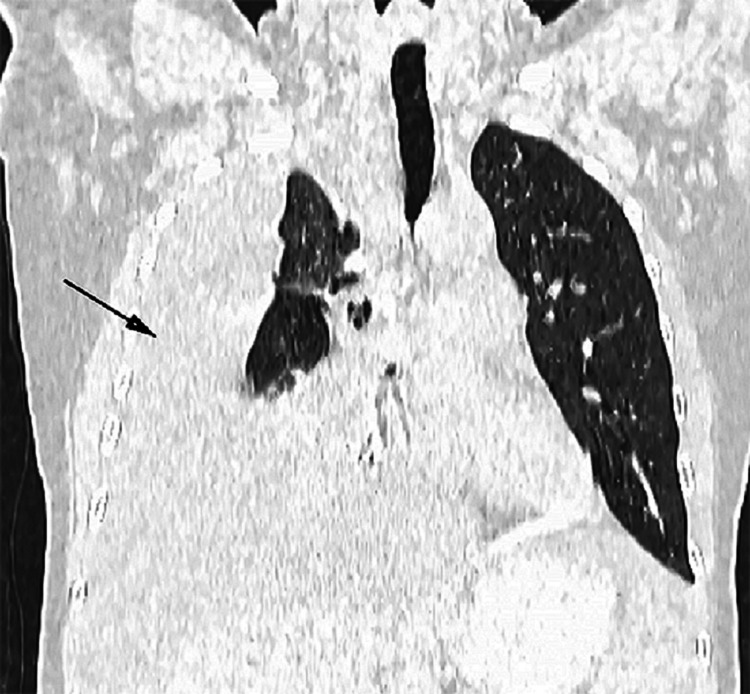
Coronal plain lung window showing the pleural effusion with atelectasis in the adjacent lung parenchyma

**Figure 4:**
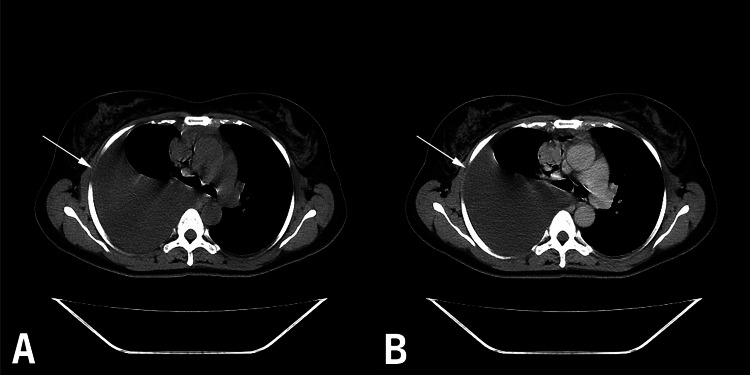
A. Plain CT (axial view) showing the pleural effusion on the right side; B. CT with contrast (axial view) showing the pleural effusion on the right side

**Figure 5:**
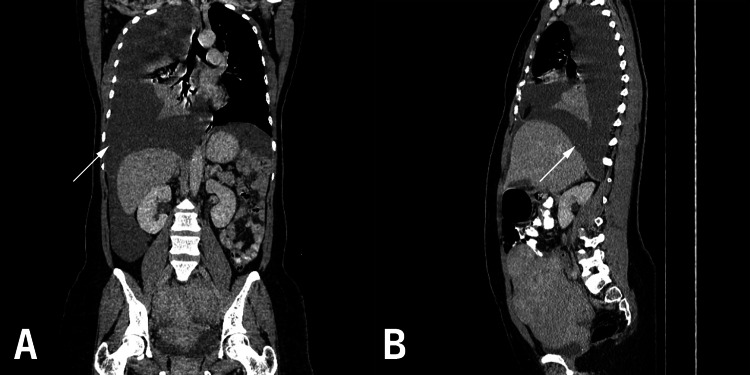
A: CT with contrast (coronal view) showing the pleural Effusion and consolidation in the adjacent lung parenchyma. Fig 5.B. CT with contrast (sagittal view) showing the pleural effusion.

Following the ultrasound findings, chest X-ray and computed tomography (CT) imaging of the chest and abdomen were performed, which showed a large, right-sided pleural effusion (Figures 1-3; Video 1) with atelectasis (Figure 4) and consolidation in the adjacent lung parenchyma (Figure 5). The pleural effusion caused a mass effect, resulting in a mild mediastinal shift to the contralateral side. Imaging also revealed a bulky uterus and focal lesions with heterogenous enhancement and calcification within, suggestive of fibroids with possible degeneration within some of them. The fibroids caused a mass effect on the adjacent structures with resultant bilateral hydroureteronephrosis. Testing also showed an elevated cancer antigen 125 (CA-125) level of 270.8 u/ml.

**Video 1:** Chest CT (axial video) showing right-sided pleural effusion

Differential diagnosis

A patient presenting with ascites, pleural effusion, and an elevated CA-125 level raises concerns of Meigs’ syndrome or pseudo-Meigs’ syndrome. However, our patient’s elevated CA-125 level (270.8 u/ml) was more suggestive of endometriosis, as it was not in the range consistent with an ovarian fibroma or malignancy. The CA-125 level, as well as the absence of an adnexal mass, led us to consider Meigs’ syndrome and pseudo-Meigs’ syndrome to be less likely than endometriosis. Lung pathologies, such as tuberculosis, pneumonia, and malignancy were also considered, but the absence of lung lesions and the transudative character of the pleural fluid made these unlikely.

Treatment

**Figure 6:**
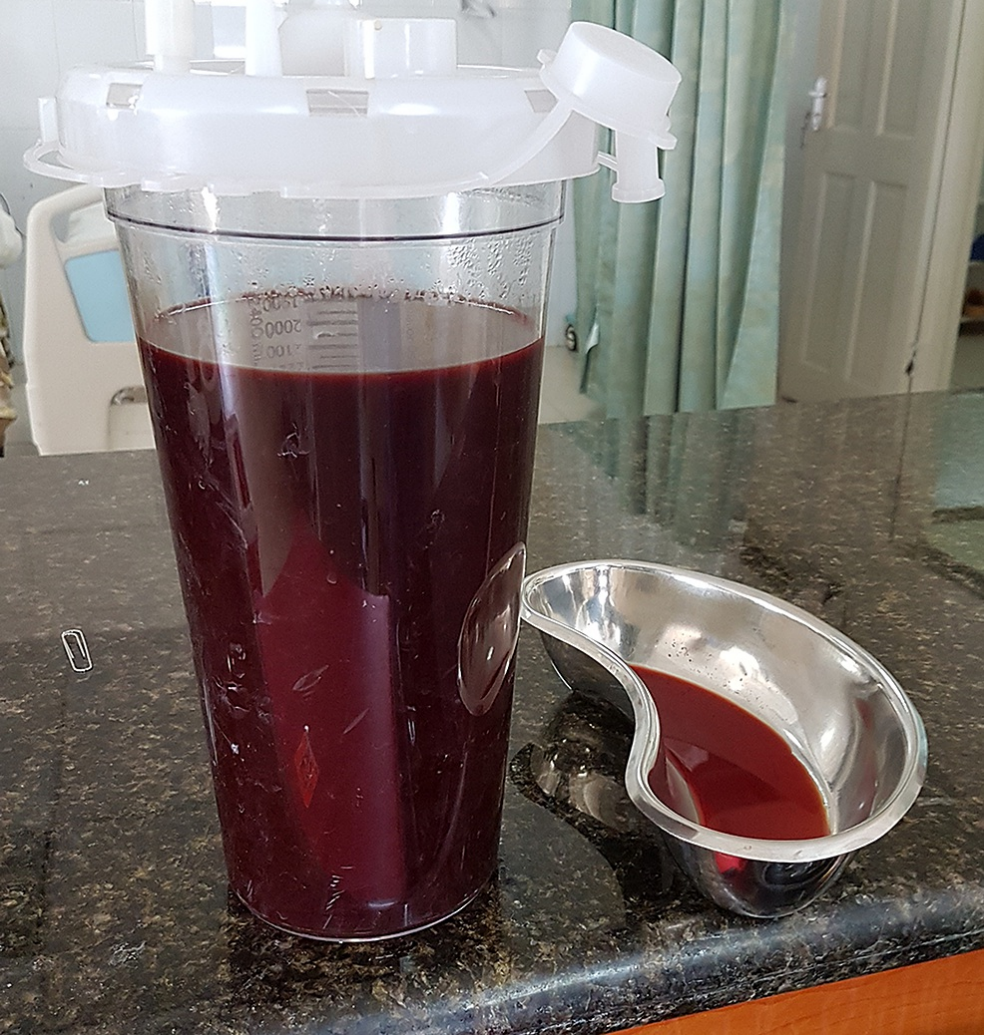
The pleural effusion drained (frankly hemorrhagic)

At the time of admission, a right pleural tap was performed, and two liters of frankly hemorrhagic fluid were drained (Figure 6). This improved her symptoms mildly, but the next day, she continued to experience dyspnoea. The pleural fluid was sent for cytological examination, however, due to limited cytologic facilities in our region, it was only determined that the sample was transudative and inadequate to rule out malignancy. While a diagnosis of thoracic endometriosis was considered, given the hemorrhagic pleural effusion, large uterine mass, ascites, and poor general condition of the patient, surgical management was deemed most appropriate.

On laparotomy, approximately 500ml of bloody fluid was found in the abdominal cavity. The uterus was enlarged, with multiple fibroids, and densely adherent to the bladder, the recto-sigmoid, caecum, and appendix. These adhesions were dissected off using sharp dissection, and a total hysterectomy with bilateral salpingo-oophorectomy was performed.

The patient recovered well after the surgery, and received injection depot medroxyprogesterone 150 mg every three months, for one year, and continued to improve with no recurrence of pleural effusion. Despite the lack of a histopathological diagnosis, due to the non-availability of special stains and immunohistochemistry, a clinical diagnosis of thoracic endometriosis syndrome was made.

Outcome and follow-up

**Figure 7:**
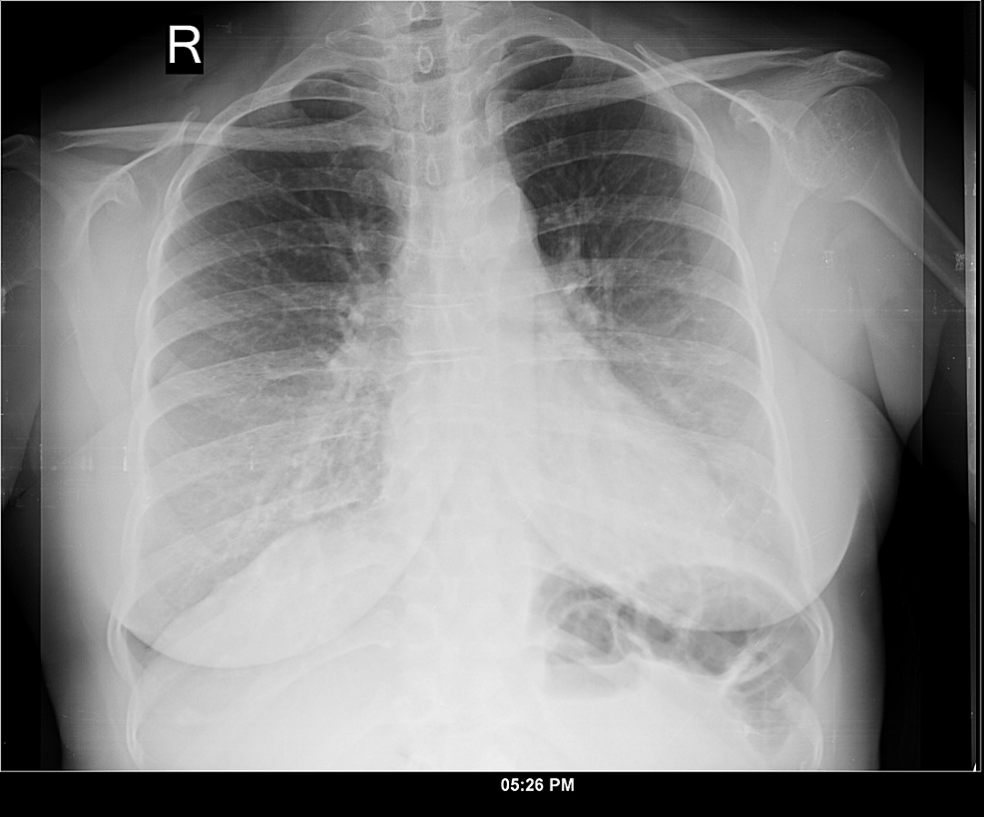
Chest X-ray posteroanterior view: taken three years after the surgery No pleural effusion is seen.

In the three years since the surgery, the patient reported no recurrence of shortness of breath, cough, or wheezing. A chest X-ray performed three years after the surgery was normal (Figure 7).

## Discussion

Endometriosis has been defined as endometrial tissue being found in sites other than the uterus [4]. The two main symptoms associated with endometriosis are pain and infertility [4]. It most commonly affects women of reproductive age, and the incidence may be as high as 50% in infertile women [5]. There is no cure for endometriosis, and numerous ways to medically and surgically manage these cases have been researched to alleviate pain and treat infertility [6].

Rarely, endometriotic tissue may be found in the lungs or pleural surface, producing a picture that we know as thoracic endometriosis syndrome. TES is a rare, underdiagnosed clinical entity that has four common clinical presentations [7]. These are catamenial pneumothorax, catamenial hemothorax, catamenial hemoptysis, and lung nodules [7]. Catamenial pneumothorax may have other causes but the cause of catamenial hemothorax is invariably thoracic endometriosis [8]. Patients with TES typically present with pneumothorax (73%), hemothorax (14%), hemoptysis (7%), and lung nodules (6%) [1].

The pathogenesis of thoracic endometriosis is not fully understood. A handful of possible explanations have been suggested such as the embolization through lymphatics and blood vessels [1], coelomic metaplasia [9], and prostaglandin-induced constriction of bronchioles and blood vessels [10]. The most compelling explanation, however, is that of retrograde menstruation leading to effluxed endometrial cells within the peritoneal cavity implanting on the diaphragmatic surface or undergoing migration to the pleural cavity [7]. Retrograde menstruation would further explain why 90% of the manifestations of TES involve right hemithorax except for nodules [1], such as in our patient, as circulating peritoneal fluid is believed to flow in a specific pattern, taking effluxed endometrial cells to the right hemidiaphragm [10].

TES is a difficult entity to diagnose due to the difficulty in establishing the temporal relationship of the features of TES with menses [2]. The dilemmas involved in the diagnosis of TES are demonstrated by intervals of up to four years between the first symptoms and diagnosis [3]. While video-assisted thoracoscopic surgery (VATS) provides the definitive diagnosis of TES [11], in most cases, the diagnosis can be made clinically [12]. Clinical diagnosis may be aided using radiographic techniques, including chest radiographs, CT scans, and magnetic resonance imaging (MRI) [13]. Non-specific radiographic findings may serve as another barrier in the use of radiographic techniques to diagnose TES [2]. Nonetheless, radiographic imaging performed at the time of menses can be compared with imaging performed midcycle, thus establishing the temporal relationship of the pathology with menses [14].

The management of TES is no less challenging than the diagnosis. The first-line management of TES involves the use of hormonal therapy to suppress ovarian estrogen secretion, using gonadotropin-releasing hormone (GnRH) analogs, oral contraceptives, progestins, aromatase inhibitors, and GnRH antagonists [13]. However, up to 60% of patients managed using medical therapy will experience a recurrence at 12 months [1]. For patients with TES who are refractory to medical therapy or with recurrent disease, a combination of surgery and postoperative hormonal therapy should be considered [11,15]. The goal of surgical management in TES is to excise all the lesions [16]. A single path should be adopted by both thoracic surgeons and gynecologists when planning surgical management of a TES patient, thus, the patient should be evaluated jointly [16]. Video thoracoscopy and laparoscopy should be executed together, to diagnose and treat patients with TES, as this would reduce hospitalization [16]. During surgery, the entire chest cavity must be carefully explored for possible lesions that may not have been detected radiologically [16]. Following surgery, some patients will still experience a recurrence of TES. However, postoperative hormonal therapy decreases this risk [16]. GnRH analogs are effective at decreasing the postoperative recurrence risk, however, other drugs such as continuous oral contraceptives have been associated with a recurrence rate of up to 33% [15]. Following surgical excision of the lesions and postoperative hormonal therapy, some patients may still have TES or a recurrence and such patients could be managed using a hysterectomy with bilateral salpingo-oophorectomy [17]. Post-surgical hormonal therapy may still be required in such patients [18]. In our case, due to the patient being unstable, and due to the lack of a definite means of excluding a malignancy causing the hemorrhagic effusion, a total hysterectomy, and bilateral salpingo-oophorectomy were performed. This was followed up by injections of depot medroxyprogesterone 150 mg every three months for one year, and the patient has remained asymptomatic for the last three years.

In conclusion, there is a need for further research to establish optimal guidelines that may aid in the diagnosis and management of TES, as the length of time to diagnosis and high recurrence rates result in significant morbidity for patients with TES. Our case demonstrates the difficulties one may encounter in diagnosing and managing a patient with TES.

## Conclusions

TES should be considered in women of childbearing age who have a pleural effusion and a history of abdominal pain. It should form part of the differential diagnosis when a malignant effusion is considered in a woman of childbearing age. As it is a condition that is hormone-responsive, progesterone preparations or gonadotropin-releasing hormone agonists may be considered if the patient is stable or as an adjunct to surgical treatment. By comparing imaging performed at the time of menses to midcycle imaging, radiographic imaging may help establish the temporal relationship in a patient with TES. Surgical management of TES should involve both thoracic surgeons and gynecologists. Postoperative hormonal therapy may be required to prevent a recurrence.
